# First Experience of Ultrasound-guided Percutaneous Ablation for Recurrent Hepatoblastoma after Liver Resection in Children

**DOI:** 10.1038/srep16805

**Published:** 2015-11-18

**Authors:** Baoxian Liu, Luyao Zhou, Guangliang Huang, Zhihai Zhong, Chunlin Jiang, Quanyuan Shan, Ming Xu, Ming Kuang, Xiaoyan Xie

**Affiliations:** 1 Department of Medical Ultrasonics, Institute of Diagnostic and Interventional Ultrasound, Division of Interventional Ultrasound, Sun Yat-Sen University, 58 Zhong Shan Road 2, Guangzhou, 510080, China; 2 Department of Pediatric Surgery, The First Affiliated Hospital of Sun Yat-sen University, Sun Yat-Sen University, 58 Zhong Shan Road 2, Guangzhou, 510080, China; 3 Department of Liver Surgery, The First Affiliated Hospital of Sun Yat-sen University, Sun Yat-Sen University, 58 Zhong Shan Road 2, Guangzhou, 510080, China

## Abstract

This study aimed to summarize the first experience with ultrasound-guided percutaneous ablation treatment (PAT) for recurrent hepatoblastoma (HB) after liver resection in children. From August 2013 to October 2014, PAT was used to treat 5 children with a total of 8 recurrent HB (mean size, 1.4 ± 0.8 cm; size range, 0.7–3.1 cm), including 4 patients with 7 tumors in the liver and 1 patient with 1 tumor in the lung. Technical success was achieved in all patients (5/5, 100%). The complete ablation rate after the first ablation session was 80% (4/5) on a patient-by-patient basis and 87.5% (7/8) on a tumor-by-tumor basis. Only 1 patient developed a fever with temperature >39 °C; it lasted 4 days after radiofrequency ablation (RFA) and was resolved by conservative therapy. During the follow-up period, new intrahepatic recurrences after PAT were detected in two patients. One died due to tumor progression 4 months after ablation. The median overall survival time after PAT was 13.8 months. PAT is a safe and promising therapy for children with recurrent HB after liver resection, and further investigation in large-scale randomized clinical trials is required to determine its role in the treatment of this disease.

Hepatoblastoma (HB) is rare; nevertheless, it is the most common primary malignant liver tumor in children younger than 5 years^1^,^2^. Multiple studies have reported HB 5-year survival rates of 60% to 87.7%^3^,^4^,^5^ with a multidisciplinary treatment approach involving chemotherapy, surgical resection and liver transplantation. Despite this improvement, the outcome for patients with recurrent HB continues to be dismal.

Recently, percutaneous ablation therapy (PAT) techniques have begun to be used for an increasing number of patients for the treatment of tumors. Among the various local ablation techniques, radiofrequency ablation (RFA) has attracted the greatest interest and is the most commonly used treatment modality for small liver cancers in adults with preserved liver function reserve and for a variety of extra-hepatic sites, including the lung^6^ and musculoskeletal system^7^. RFA has been reported to achieve a complete response comparable to the response obtained with surgical liver resection with a lower morbidity rate of 5–18%^8^,^9^,^10^ and a lower mortality rate of 0–1.4%^10^,^11^,^12^. Percutaneous ethanol injection (PEI) is also an effective treatment that has been widely used in patients with small tumors. Although PEI is inferior to RFA in its local control ability, it is useful for the treatment of tumors in high-risk locations.

However, PAT achieves exceptional response in children. We hypothesized that PAT is an alternative treatment for recurrent HB and could serve as an adjunct to the existing treatment modalities for HB. Therefore, the aim of our study was to summarize and report our first experience of PAT for recurrent HB in children.

## Patients and Methods

### Patients

This retrospective study was performed according to the guidelines of the Helsinki Declaration. It was registered and approved by the ethics committee at The First Affiliated Hospital of Sun Yat-sen University. All patients’ parents gave written informed consent before treatment.

From August 2013 to October 2014, postoperative recurrences were detected in a total of 5 children with HB. All patients underwent PAT for their recurrent tumors. We retrospectively reviewed their records. The patient’s ages ranged from 23 months to 11 years (mean age, 4 years), andthere were 3 males and 2 females. The initial tumor size in these patients was 7.1 ± 3.0 cm (range, 2.4–12.8 cm). According to the International Society of Pediatric Oncology (SIOPEL) study group, 2 patients were staged as Pre Treatment Extent of Disease (PRETEXT) stage II, 2 were staged as PRETEXT stage III and 1 was staged as PRETEXT stage IV. Before surgery, 3 patients received neoadjuvant chemotherapy and 1 patient received TACE plus neoadjuvant chemotherapy. The diagnosis of suspected HB before chemotherapy was confirmed by biopsy. All patients underwent anatomic liver resections, including left hemihepatectomy in 2 patients, left trisectionectomy 1 patient and segmentectomy in 1 patient. The remaining patient underwent non-anatomic liver resection due to almost the entire tumor protruding the capsule. Moreover, all patients received postoperative chemotherapy. After a follow-up of 2 to 73 months, a total of 8 recurrent tumors were detected in the 5 enrolled patients, including 4 patients with 7 recurrences in the liver and 1 patient with 1 recurrence in the lung. The diagnosis of recurrence was based on the observation of newly emerged tumors on computed tomography (CT) and/or color Doppler ultrasound (US) images and an increase in serum alpha-fetoprotein (AFP). The mean diameter of the recurrent HBs was 1.4 ± 0.8 cm (range, 0.7–3.1 cm). The detailed baseline characteristics of all 5 patients are listed in Table 1, and Table 2 shows the serum AFP levels for these patients at multiple time points.

### Percutaneous Ablation Procedures

According to our protocol, RFA was recommended as the first choice for the treatment of favorable tumors, and PEI was performed for unfavorable tumors containing a capsule. Unfavorable tumors were defined as tumors located <5 mm from important structures, such as the bowel, bile duct, and liver capsule. All ablation procedures were guided and monitored by US. US was performed by X.Y.X., who had 25 years of experience with US. All patients were hospitalized prior to treatment.

### Radiofrequency Ablation

RFA was performed under general anesthesia. RFA was performed using a Cool-tip™ RFA system (Valleylab, Boulder, CO, USA), which consisted of a RF generator with a maximum power of 200 Wand a 17-gauge internally cooled electrode. Using US guidance, the radiofrequency electrode was carefully introduced into the target lesion at the predetermined location. The number of electrodes was dependent on the tumor size, shape, and location. If necessary, after the first application, the needle was pulled out by 1 cm and a second application was started. After the ablation was completed, the needle track was carefully treated with the electrode being retracted by 1 cm increments to prevent bleeding and tumor seeding. A single treatment session consisted of one or two applications. A safety margin of 0.5 cm was employed during the RFA procedure.

### Percutaneous Ethanol Injection

After the administration of general anesthesia, an 18-gauge needle (Hakko Co., Ltd, Nagano, Japan) was inserted into the center of the tumor nodule under US guidance, and the tip of the needle was positioned at the inferior aspect of the tumor. Ethanol was injected until the entire tumor appeared hyperechoic upon performing the pullback technique. Two or three cycles of PEI were performed per week, depending on patient’s tolerance.

### Treatment Outcomes and follow-up

One month after the treatment of recurrent HB, contrast-enhanced CT was performed to evaluate local efficacy. Complete ablation was defined as complete non-enhancement of the treated lesion on contrast-enhanced CT^13^,^14^. In cases with a viable residual tumor, additional ablation was performed with the aim of complete ablation. If the tumor was still viable after additional ablation, ablation therapy was considered a failure.

All patients underwent post-PAT chemotherapy. During the follow-up period, the serum AFP level was measured, and color Doppler US was performed every month. If necessary, additional contrast-enhanced CT was performed. Assessment of response to the treatment was performed with contrast-enhanced CT and AFP level. The definitions used were as follow: complete response = no evidence of disease after complete ablation and normal AFP level; partial response = any tumor size decrease associated with decreased AFP level (>1 log less than the original value); stable disease = no change in tumor size and no change or less than 1 log decrease of AFP level; and progressive disease = unequivocal increase in size of the tumor size and/or new recurrence and/or any unequivocal increase in AFP level^15^.

### Statistical Analysis

The statistical analyses were performed using SPSS 16.0 statistical software (Chicago, IL, USA). All data are reported as the mean ± standard deviation. Student’s t test or the Mann-Whitney test was used to assess the differences between two groups of quantitative variables. The disease-free survival (DFS) and cumulative overall survival (OS) were assessed using the Kaplan-Meier method. The disease-free time was defined as the period from the date of PAT to the development of a new recurrence, death or last follow-up. The survival time after ablation was defined as the period from the completion of PAT to death or last follow-up. The survival time after diagnosis was defined as the period from the diagnosis of HB to death or last follow-up.

## Results

### Tumor Response

The ablation procedures and outcomes of all 5 enrolled patients are shown in Table 3. RFA was performed in 3 children (No. 1, 4 and 5) with 4 tumors, including 3 tumors in the liver and 1 tumor in the lung. The mean ablation time per tumor was 12.8 min (range, 9–20 min). All tumors were completely ablated as indicated by the contrast-enhanced CT results 1 month after PAT.

**Figure 1:**
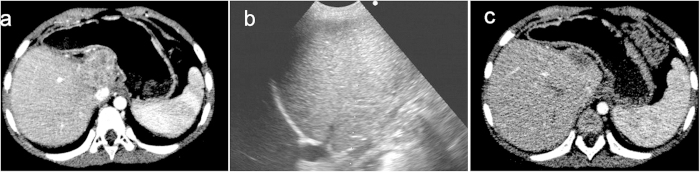
A 4-year-old girl with recurrent hepatoblastoma after initial liver resection. Contrast-enhanced computer tomography (CT) shows a 3.1 cm recurrent tumor in the caudal lobe (**a**). After percutaneous ethanol injection (**b**), contrast-enhanced CT shows disappearance of the enhancement within the treated tumor (**c**).

PEI was performed in another 2 children (No. 2 and 3) with 4 tumors in the liver that were difficult to ablate using RFA (1 patient with a tumor in the caudate lobe adjacent to the stomach and 3 tumors in segment 6 close to the liver capsule). The mean volume of injected ethanol per tumor was 5.8 mL (range, 3–8 mL). One month later, a residual tumor was detected, and it was treated with an additional injection of 5 mL of ethanol. Finally, complete ablation was achieved after a second session of PAT. The remaining tumors achieved complete ablation after the first ablation (Fig. 1).

**Figure 2:**
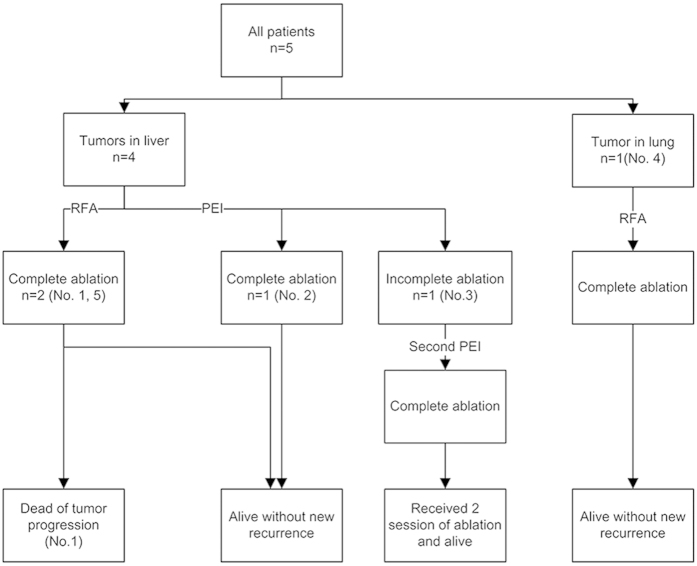
Treatment flow chart.

Therefore, technical success of PAT for recurrent HB was achieved in all patients (5/5, 100%) (Fig. 2). The complete ablation rate after the first ablation session was 80% (4/5) on a patient-by-patient basis and 87.5% (7/8) on a tumor-by-tumor basis. For the RFA technique, the complete ablation rate after the first session was 100% on both a patient-by-patient basis (3/3) and a tumor-by-tumor basis (4/4). For the PEI technique, the complete ablation rate after the first session was 50% (1/2) on a patient-by-patient basis and 75% (3/4) on a tumor-by-tumor basis.

All patients received chemotherapy after PAT. The following chemotherapeutic regimens were used: irinotecan plus vincristine plus nedaplatin for two patients (No.1 and 2); irinotecan plus vincristine plus temozolomide for two patients (No. 3 and 4); and pirarubicin plus nedaplatin plus cyclophosphamide for 1 patient (No. 5).

### Adverse Events and Complications of PAT

No patient died during the ablation treatment. Four patients with recurrent tumors in the liver showed evaluated aminotransferase levels after PAT and the levels returned to normal within 1 to 2 weeks. Only 1 patient (No. 2) developed a fever with a temperature >39 °C; it lasted for 4 days after RFA, and was resolved by conservative therapy. There were no signs of hemorrhagic accidents, pneumothorax, infection or damage to adjacent organs such as the gallbladder, bile duct, bowels, or stomach, after ablation. No skin burns were observed after RFA.

### Follow-up and Survival

After a follow-up of 5–16 months, new distant intrahepatic recurrences, without extrahepatic recurrences, were detected in two patients (No. 1 and 3). One patient (No. 1) received chemotherapy treatment alone. However, that patient’s AFP level did not decrease, and she died due to tumor progression 4 months after ablation. The other patient (No. 3) received 2 sessions of ablation for the new recurrences, and he survived for 9 months after ablation and 82 months after HB diagnosis. One patient (No. 4) was considered to have a partial response because her AFP level decreased but remained abnormal. A complete response was achieved in the remaining 2 patients (No. 2 and 5), and they survived for 7 and 16 months, respectively, after ablation (Table 2). Thus, the overall survival rate was 80% (4/5) at the end of follow-up (Fig. 2).

**Figure 3:**
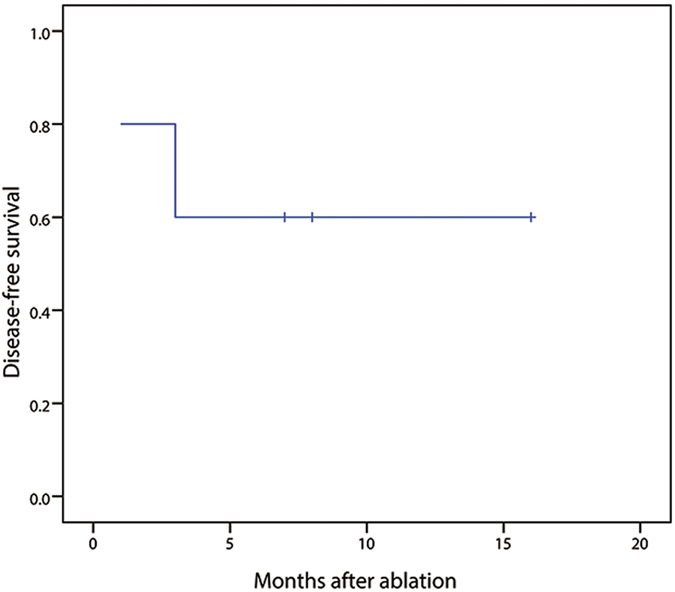
Disease-free survival curve of patients after PAT.

The median DFS time after ablation was 10.4 months, and the DFS rate at 6 months was 60% (Fig. 3). The median cumulative OS time after ablation was 13.8 months, whereas the median cumulative OS time after HB diagnosis was 68.2 months.

## Discussion

HB is the most common malignant liver tumor that occurs during childhood^1^,^2^. Advancements in chemotherapeutic agents and administration protocols as well as innovations in surgical approaches, including the use of vascular exclusion, ultrasonic dissection techniques and liver transplantation, have greatly improved the outcome of children with HB. However, challenges remain in the management of patients with recurrent HB^1^. For example, although liver transplantation is a successful treatment option for children with unresectable HB, with primary transplantation having a 90% survival rate, it has a poor survival rate for patients with recurrent HB after previous liver resection^2^,^16^. Therefore, further understanding of the optimal treatment is necessary for the management of these cases.

Different treatment modalities have been applied for recurrent HB. Chemotherapy is necessary for patients with recurrent HB after surgery. The study of Marsh *et al.*^17^ demonstrated that the combined use of sorafenib and bevacizumab was effective for the treatment of recurrent HB that had previously been extensively treated with multiple chemotherapeutic regimens. In that study, the patient had a progression-free survival of 3 months. However, local ablation helps enable chemotherapy to destroy micrometastases^18^. Considering the age and nutritional state of the child, repeated liver resection was considered difficult, even impossible. Although liver transplantation is an accepted therapy for children with recurrent or refractory HB^2^,^16^, it is limited by donor availability. If a liver can become tumor free by PAT, it should be considered as a possible alternative treatment. To date, no studies on the efficacy of PAT have been conducted in the particular setting of recurrent HB, although recent case reports have confirmed an acceptable safety profile of PAT in that setting. In 2008, Ye *et al.*^14^ reported a 2-year-old boy with recurrent HB after liver resection treated by percutaneous RFA, and there was no imaging evidence of recurrence after a follow-up of 2 years. Recently, Dunn *et al.*^19^ demonstrated another successful treatment of pulmonary metastasis of HB in a 2-year-old boy using RFA. Both research groups deemed RFA to be a promising technique in children with recurrent HB. Here, we summarized the first experience of US-guided PAT for recurrent HB in children in a tertiary hospital. Three patients were treated by RFA, and 2 patients were treated by PEI, and no patients died due to the ablation treatment. The overall survival rate was 80% at the end of follow-up. Among the 5 enrolled patients, 3 were alive without new recurrence at the end of follow-up. PAT may add a new dimension to the treatment of recurrent HB.

We applied RFA and PEI for the treatment of recurrent tumors. RFA is recommended as the first choice for the majority of tumors, except tumors located <5 mm from important structures, such as the bowel, bile duct, and liver capsule. For hepatocellular carcinoma in adults, prospective randomized trials^20^,^21^ have revealed that RFA yields higher rates of complete tumor necrosis with fewer treatment sessions and better survival compared with PEI. In our case series, complete ablation was achieved in all patients after the first session of RFA, whereas 1 residual tumor was detected after initial PEI. Although 1 patient developed a fever with a temperature >39 °C after RFA, no major complications occurred. Further large-scale randomized clinical trials are required to compare RFA with PEI for the treatment of children with recurrent HB.

Four tumors in 2 patients were treated by PEI. Close attention should be paid to the amount of ethanol administered to children during PEI. It is known that for tumors of 3.0 cm or smaller in diameter, the volume of injected ethanol should be calculated as the volume of an oversized sphere, as follows^22^: V = 4/3π [(D/2 + 0.5)^3^], where V is the volume in milliliters, and D is the largest tumor dimension in centimeters. For tumors that are 3.1–5.0 cm in diameter, the least amount of ethanol that should be administered is equal to the volume of the tumor (V = 4/3π [(D/2)^3^]), and the upper limit of ethanol volume per session to minimize the toxic effects of ethanol to the adult patient is 60 mL. That is to say, the total amount of ethanol required for the complete destruction of a tumor lesion is approximately 4 mL for a lesion of 1 cm in diameter, 14 mL for a lesion of 2 cm in diameter, and 33 mL for a lesion of 3 cm in diameter^23^. A sufficient dose of ethanol is necessary to achieve necrosis of the entire tumor.

In contrast, a high-dose of ethanol may induce a choking sensation, cough, tachycardia, and even respiratory depression in adults^22^, and the toxic effects of ethanol are greater and more complex in children. Therefore, the dosage protocol used for children required further evaluation to achieve an appropriate balance between local effectiveness and avoidance of complications.

As expected, there is very limited experience of ablation in children. Recently, Gomez *et al.*^24^ searched MEDLINE for papers published between 1995 and 2012 that reported the outcomes of PAT for patients less than 18 years of age. They only found the following numbers of patents reported to have undergone ablation: 3 for liver lesions, 9 for lung metastases, 11 for bone and/or soft tissue lesions and 4 for kidney or pancreatic lesions. Based on the available data, our case series seems to have an acceptable sample size to summarize and evaluate our treatment experience. Furthermore, in addition to recurrent HB, PAT seems to be feasible in children with primary, recurrent or metastatic tumors in various organs. In those children treated by ablation, only 6 (21%) required treatment other than supportive care due to a complication. In the 26 patients whose survival after PAT was reported, the mean overall survival was 22 months (median: 18 months; range: 0.7–96 months).

There are several limitations to the present study. First, only 5 patients with recurrent HB after hepatectomy who underwent PAT were enrolled in this study. However, recurrent HB is rare and it is difficult to accumulate a large sample size of patients with this condition. Secondly, this was a retrospective data analysis from a single institution. Therefore, a properly designed prospective multicenter study is needed to determine if this technique does in fact have efficacy in this setting. Third, this was a single arm PAT treatment study, and there was no direct comparison to other treatment modalities. Randomized controlled clinical trials are needed to provide a comparative evaluation of this technique for the treatment of recurrent HB. Moreover, the follow-up period was short. The long-term results of this treatment are still unclear.

In conclusion, our experience with these 5 cases, although small in number, suggests the feasibility of PAT for recurrent HB. PAT may be a good adjunct to the existing treatment modalities for HB. However, PAT for pediatric tumors is still in its infancy, and further investigation in large-scale randomized clinical trials is necessary to confirm our observations and further determine the role of PAT.

## Additional Information

**How to cite this article**: Liu, B. *et al.* First Experience of Ultrasound-guided Percutaneous Ablation for Recurrent Hepatoblastoma after Liver Resection in Children. *Sci. Rep.* **5**, 16805; doi: 10.1038/srep16805 (2015).

## Figures and Tables

**Table 1:** Baseline characteristics of all 5 patients.

Patient No./Age/gender	PRETEXT stage	Tumor size at diagnosis (cm)	Pre-surgery treatments	Tumor size after chemotherapy (cm)	Involved Couinaud segments at the time of surgery	Surgery	Post-surgery chemotherapy	Interval time between surgery and recurrences (months)	Location of recurrences	No. of recurrence	Maximum size in diameter of recurrences (cm)	The site of tumor recurrence in the liver
1/2y6m/F	II	9 × 8 × 8	Chemotherapy	3.8 × 2.6 × 5.3	S2.3.4	Left hemihepatectomy	Yes	4	Liver	2	1.2–2.1	S6
2/4y/F	IV	9.4 × 7.4 × 5.1, 5.1 × 3.9 × 3.8	Chemotherapy	6.0 × 4.5, 2.2 × 1.5	S4/S4.8	Left trisectionectomy	Yes	11	Liver	1	3.1	S1
3/11y/M	II	8.6 × 5.7 × 6.2	None	–	S5.6	Tumor resection	Yes	73	Liver	3	0.7–1.5	S6
4/1y11m/F	III	8.9 × 7.1 × 7.0	Chemotherapy, TACE	6.9 × 5.6 × 5.4	S2.4	Left hemihepatectomy	Yes	2	Lung	1	1.0	–
5/3y3m/M	III	9.8 × 8.1 × 7.7	Chemotherapy	6.2 × 6.0 × 5.2	S4	Segmentectomy	Yes	9	Liver	1	1.0	S5

**Table 2:** Serum AFP levels for all the 5 patients at multiple time points.

Patient No.	AFP values (ng/mL)			
	At diagnosis	Post-resection	At recurrence	Post-ablation
1	518924.8	45.07	18971.24	44891.32
2	>999000	4.52	15.73	3.21
3	39813.28	826.06	11507.64	691.26
4	34346.1	35	1082.95	19.64
5	34128.5	14.71	543.2	6.72

**Table 3:** Ablation procedures and outcomes of all the 5 enrolled patients.

Patient No.	Ablation technique	Sessions of ablation	Outcome of ablation	Adverse events and complications of ablation	Treatment response	Recurrence after ablation	Interval time between ablation and recurrence (months)	Treatments for recurrence after ablation	Survival time after ablation (months)	Survival time after diagnosis (months)	Outcome at Last Follow-up (alive or dead/cause of death)
1	RFA	1	Complete	Fever	PD	Yes	1	Chemotherapy	5	13	Dead/disease progression
2	PEI	1	Complete	—	CR	No	—	—	16	31	Alive
3	PEI	2	Complete	—	PD	Yes	3	PEI, RFA, chemotherapy	9	82	Alive
4	RFA	1	Complete	—	PR	No	—	—	7	19	Alive
5	RFA	1	Complete	—	CR	No	—	—	8	21	Alive

PEI: percutaneous ethanol injection; RFA: radiofrequency ablation.

CR: complete response, PR: partial response, SD: stable disease, PD: progressive disease.

## References

[b1] Kremer N., Walther A. E. & Tiao G. M. Management of hepatoblastoma: an update. Curr Opin Pediatr 26, 362–369 (2014). 24759227 10.1097/MOP.0000000000000081

[b2] Koneru B. *et al.* Liver transplantation for hepatoblastoma. The American experience. Ann Surg 213, 118–121 (1991). 1847033 10.1097/00000658-199102000-00004 PMC1358382

[b3] Horton J. D., Lee S., Brown S. R., Bader J. & Meier D. E. Survival trends in children with hepatoblastoma. Pediatr Surg Int 25, 407–412 (2009). 19308432 10.1007/s00383-009-2349-3

[b4] Tannuri A. C., Cristofani L. M., Teixeira R. A., Odone Filho V. & Tannuri U. New concepts and outcomes for children with hepatoblastoma based on the experience of a tertiary center over the last 21 years. Clinics (Sao Paulo) 70, 387–392 (2015). 26106955 10.6061/clinics/2015(06)01 PMC4462574

[b5] Qiao G. L. *et al.* Predictors of survival after resection of children with hepatoblastoma: A single Asian center experience. Eur J Surg Oncol 40, 1533–1539 (2014). 25103357 10.1016/j.ejso.2014.07.033

[b6] Simon C. J. *et al.* Pulmonary radiofrequency ablation: long-term safety and efficacy in 153 patients. Radiology 243, 268–275 (2007). 17392258 10.1148/radiol.2431060088

[b7] Kim S. J. *et al.* Ultrasonography-guided radiofrequency ablation of malignant musculoskeletal soft-tissue tumors using the “moving-shot” technique at a single-institution experience. Ultrasound Q 30, 295–300 (2014). 25415868 10.1097/RUQ.0000000000000062

[b8] Guglielmi A. *et al.* Radiofrequency ablation versus surgical resection for the treatment of hepatocellular carcinoma in cirrhosis. J Gastrointest Surg 12, 192–198 (2008). 17999123 10.1007/s11605-007-0392-8

[b9] Ogihara M., Wong L. L. & Machi J. Radiofrequency ablation versus surgical resection for single nodule hepatocellular carcinoma: long-term outcomes. HPB (Oxford) 7, 214–221 (2005). 18333193 10.1080/13651820510028846 PMC2023955

[b10] Livraghi T. *et al.* Treatment of focal liver tumors with percutaneous radio-frequency ablation: complications encountered in a multicenter study. Radiology 226, 441–451 (2003). 12563138 10.1148/radiol.2262012198

[b11] Ding J. *et al.* Complications of thermal ablation of hepatic tumours: comparison of radiofrequency and microwave ablative techniques. Clin Radiol 68, 608–615 (2013). 23399463 10.1016/j.crad.2012.12.008

[b12] Parisi A. *et al.* Liver resection versus radiofrequency ablation in the treatment of cirrhotic patients with hepatocellular carcinoma. Hepatobiliary Pancreat Dis Int 12, 270–277 (2013). 23742772 10.1016/s1499-3872(13)60044-2

[b13] Wang S. *et al.* First experience of high-intensity focused ultrasound combined with transcatheter arterial embolization as local control for hepatoblastoma. Hepatology 59, 170–177 (2014). 23813416 10.1002/hep.26595

[b14] Ye J., Shu Q., Li M. & Jiang T. A. Percutaneous radiofrequency ablation for treatment of hepatoblastoma recurrence. Pediatr Radiol 38, 1021–1023 (2008). 18535824 10.1007/s00247-008-0911-0

[b15] Moon S. B., Shin H. B., Seo J. M. & Lee S. K. Hepatoblastoma: 15-year experience and role of surgical treatment. J Korean Surg Soc 81, 134–140 (2011). 22066113 10.4174/jkss.2011.81.2.134 PMC3204570

[b16] Browne M. *et al.* Survival after liver transplantation for hepatoblastoma: a 2-center experience. J Pediatr Surg 43, 1973–1981 (2008). 18970927 10.1016/j.jpedsurg.2008.05.031

[b17] Marsh A. M., Lo L., Cohen R. A. & Feusner J. H. Sorafenib and bevacizumab for recurrent metastatic hepatoblastoma: stable radiographic disease with decreased AFP. Pediatr Blood Cancer 59, 939–940 (2012). 22492703 10.1002/pbc.24171

[b18] Frezza E. E. Therapeutic management algorithm in cirrhotic and noncirrhotic patients in primary or secondary liver masses. Dig Dis Sci 49, 866–871 (2004). 15259511 10.1023/b:ddas.0000030101.81734.47

[b19] Dunn C. L., Lucas J. T. Jr., Clark H. & McLean T. W. Successful radiofrequency ablation for recurrent pulmonary hepatoblastoma. Pediatr Blood Cancer, 10.1002/pbc.25617 (2015). PMC9134866 26109368

[b20] Lencioni R. A. *et al.* Small hepatocellular carcinoma in cirrhosis: randomized comparison of radio-frequency thermal ablation versus percutaneous ethanol injection. Radiology 228, 235–240 (2003). 12759473 10.1148/radiol.2281020718

[b21] Orlando A., Leandro G., Olivo M., Andriulli A. & Cottone M. Radiofrequency thermal ablation vs. percutaneous ethanol injection for small hepatocellular carcinoma in cirrhosis: meta-analysis of randomized controlled trials. Am J Gastroenterol 104, 514–524 (2009). 19174803 10.1038/ajg.2008.80

[b22] Kuang M. *et al.* Ethanol Ablation of Hepatocellular Carcinoma Up to 5.0 cm by Using a Multipronged Injection Needle with High-Dose Strategy. Radiology 253, 552–561 (2009). 19709992 10.1148/radiol.2532082021

[b23] Hara F. *et al.* A child with adrenocortical carcinoma who underwent percutaneous ethanol injection therapy for liver metastasis. J Pediatr Surg 38, 1237–1240 (2003). 12891501 10.1016/s0022-3468(03)00276-8

[b24] Gomez F. M., Patel P. A., Stuart S. & Roebuck D. J. Systematic review of ablation techniques for the treatment of malignant or aggressive benign lesions in children. Pediatr Radiol 44, 1281–1289 (2014). 24821394 10.1007/s00247-014-3001-5

