# Corrigendum: First Experience of Ultrasound-guided Percutaneous Ablation for Recurrent Hepatoblastoma after Liver Resection in Children

**DOI:** 10.1038/srep33424

**Published:** 2016-09-16

**Authors:** Baoxian Liu, Luyao Zhou, Guangliang Huang, Zhihai Zhong, Chunlin Jiang, Quanyuan Shan, Ming Xu, Ming Kuang, Xiaoyan Xie

Scientific Reports 5: Article number: 16805; 10.1038/srep16805 published online: 11 18 2015; updated: 09 16 2016.

In the original version of this Article, Affiliation 1 was incorrectly given as ‘Department of Medical Ultrasonics, Institute of Diagnostic and Interventional Ultrasound, Division of Interventional Ultrasound, Sun Yat-Sen University, 58 Zhong Shan Road 2, Guangzhou, 510080, China’. The correct affiliation is listed below:

Department of Medical Ultrasonics, Institute of Diagnostic and Interventional Ultrasound, Division of Interventional Ultrasound, The First Affiliated Hospital of Sun Yat-sen University, Sun Yat-Sen University, 58 Zhong Shan Road 2, Guangzhou, 510080, China.

This error has now been corrected in the PDF and HTML versions of this Article.

